# Effects and Mechanisms of *Lactobacillus rhamnosus* MP108 Probiotic Intervention During Pregnancy on Offspring Neurodevelopment

**DOI:** 10.1155/ijfo/5160096

**Published:** 2026-04-27

**Authors:** Weizhi Zhong, Lilin Xiao, Huilan Ye, Qiaoyi Zhang, Yu Gao, Jiaqi Wang, Lina Pan, Cailing Chen, Wei Li

**Affiliations:** ^1^ Department of Basic Research, Global Research and Development Center, Ausnutria Dairy (China) Co., Ltd., Changsha, China; ^2^ National Center of Technology Innovation for Dairy, Hohhot, China

**Keywords:** gut microbiota, *Lactobacillus rhamnosus* MP108, metabolomics, neurodevelopment, prenatal probiotics

## Abstract

The early stages of life constitute a crucial period for neurodevelopment, during which maternal gut microbiota dysbiosis can impact offspring neurodevelopment via the microbiota–gut–brain axis. This study aimed to examine the ameliorative effects of administering the probiotic *Lactobacillus rhamnosus* MP108 during pregnancy on neurodevelopmental impairments in offspring induced by a maternal high‐fat diet. The findings indicated that the MP108 intervention significantly ameliorated cognitive and behavioral abnormalities in offspring resulting from maternal obesity. Additionally, it restructured the gut microbiota composition in both mothers and offspring, enhancing the prevalence of beneficial bacteria such as Blautia and Alloprevotella. Metabolomic analysis further revealed that MP108 reduced intestinal levels of L‐homocystine and methionine while increasing those of L‐(+)‐arginine, L‐(+)‐citrulline, and L‐tyrosine. Furthermore, MP108 markedly attenuated the aberrant expression of neuroinflammatory and myelin formation–related genes in the hippocampal region of offspring, including cyclooxygenase‐2 (Cox2), nuclear factor kappa‐B (NF‐κB), and myelin basic protein (MBP), thereby restoring their expression levels to normative ranges. This study represents the first evidence that maternal intervention with MP108 during gestation influences gut microbiota composition and metabolite profiles, subsequently reducing neuroinflammation and facilitating neural development. This mechanism effectively alleviates the detrimental effects of a maternal high‐fat diet on the neural development of offspring, thereby providing empirical support for the potential application of MP108 in the prevention and amelioration of neurodevelopmental disorders.

## 1. Introduction

The early stages of life are crucial for neurodevelopment, during which the host gut microbiota plays a significant regulatory role. Disruptions in maternal gut microbiota can impair neurodevelopment in infants [1]. Recently, the microbiota–gut–brain axis has been recognized as a key pathway connecting gut microbiota to neurodevelopment and metabolic balance, with its dysfunction linked to various neurodevelopmental disorders [2].

Factors such as high‐fat diets (HFDs) during pregnancy, depression, anxiety, and nutrient deficiencies can cause gut microbiota dysbiosis [3]. Among these, obesity—a growing public health issue—has garnered attention for its effects on both mothers and their children. Epidemiological and genetic evidence suggest that the maternal gut microbiota exerts a significant influence on offspring through two primary pathways. Firstly, during pregnancy, the maternal gut microbiota provides essential metabolites and substrates for fetal growth via metabolic transfer, which facilitates the proliferation and maturation of central and peripheral immune cells and the formation of neural circuits. Secondly, during delivery and the early postnatal period, the vertical transmission of maternal microbiota offers robust immune stimulation to the offspring, thereby inducing transcriptional programs that are critical for metabolism and development. Additionally, maternal gut dysbiosis, which may result from dietary modifications or antimicrobial treatments, affects the offspring’s microbiome, brain function, and behavior [4].

Previous research has demonstrated that maternal dietary fiber intake can ameliorate behavioral dysfunction by modulating gut microbiota composition and short‐chain fatty acid (SCFA) production [5]. However, the impacts and underlying mechanisms of probiotic interventions during pregnancy on offspring neurodevelopment remain inadequately understood. The potential of maternal probiotic consumption to ameliorate adverse outcomes in offspring through modulation of the microbiota–gut–brain axis requires further validation.

In this study, administration of probiotics to obese pregnant mouse models revealed that maternal probiotic intake during gestation can alleviate neurodevelopmental impairments in offspring induced by maternal obesity. These findings offer significant evidence supporting the role of the microbiota and their metabolites in the regulation of emotional states and related neural plasticity. Additionally, this research lays a theoretical groundwork for the development of functional foods and clinical interventions in the future.

## 2. Materials and Methods

### 2.1. Probiotic Cultivation

*Lactobacillus rhamnosus* MP108 was provided by Jinqi Biotechnology. In a laminar flow hood, *Lactobacillus rhamnosus* MP108 was streaked from the stock culture tube onto MRS solid medium and incubated at 37°C for 10 h. A 2% (v/v) inoculum was then transferred to a test tube containing 5 mL of MRS liquid medium for further cultivation. Based on the growth curve, fermented broth from the late logarithmic growth phase was collected. The culture was centrifuged (4°C, 6000 × *g*, 10 min) to obtain the bacterial pellet. The pellet was washed twice with sterile physiological saline. After washing, 30% glycerol was added and thoroughly mixed. The mixture was stored at −80°C for future use. Before use, wash twice with sterile saline. Resuspend in saline at the appropriate dilution factor to achieve a live bacterial concentration of 10^9^ CFU/mL.

### 2.2. Animal Experiment

This experiment utilized C57BL/6J mice, aged 6 weeks, weighing approximately 18–20 g, and of SPF grade. The mice were purchased from Beijing VTLH Laboratory Animal Co., Ltd. and housed at the Experimental Animal Center of Jiangnan University. The study was approved by the Ethics Committee of the Experimental Animal Management Center at Jiangnan University (Approval No.: JN.NO20240315c0450825[083]). Mouse housing strictly adhered to ethical guidelines for animal experimentation. All mice were maintained under conditions of 21°C–25°C ambient temperature and 40%–60% humidity, with a 12‐h light–12‐h dark cycle. Following a one‐week acclimation period, mice were randomly assigned to groups based on body weight.

The specific steps of the feeding protocol are as follows: Before the experiment, female rats underwent a one‐week acclimation period and were then randomly divided into two groups. One group (*n* = 6) was fed a standard diet, while the other group (*n* = 18) received a HFD. Male rats were uniformly fed the standard diet. The standard diet group received a laboratory‐provided diet containing 10%–14% fat calories, while the HFD group was fed a diet containing 60% fat calories (TP23300, Nantong Trofi). Dietary intervention commenced 5 weeks before conception and continued throughout gestation and lactation. Subsequently, male and female rats were housed together in a 2:1 ratio for free mating, with external disturbances minimized during the mating period. The onset of pregnancy was confirmed via vaginal plug detection. After pregnancy, pregnant mice were randomly divided into three groups for further experiments: the control group (6 mice) was fed a conventional diet and administered 0.2 mL of sterile saline solution via gastric gavage daily; the model group (6) fed a HFD and administered 0.2 mL sterile saline via daily gastric lavage; the probiotic intervention group (12) fed a HFD and administered MP108 bacterial preparation at 4 × 10^9^ CFU/mL via daily gastric lavage. After 3 weeks of gestation, pregnant rats gave birth naturally. All offspring were nursed by their mothers for 3 weeks before weaning. Mothers in the HFD group were switched to conventional diet, while all offspring continued on conventional diet. Offspring were divided into three groups based on maternal intervention. At Week 3, all offspring underwent open‐field behavioral testing and novel object recognition (NOR) testing to assess the effects of probiotics on offspring behavior resulting from maternal HFD exposure. The specific feeding protocols are detailed in Table 1.

**TABLE 1:** Mouse experimental groups and housing methods.

Daily treatment protocol for mother mice	Offspring mouse groups
Normal diet + 0.2 mL saline gavage during pregnancy (Weeks 6–8)	Control group (CD)
11 weeks high‐fat diet + 0.2 mL saline gavage during pregnancy (Weeks 6–8)	High‐fat diet (HFD)
High‐fat diet for 11 weeks + gestational gavage of 0.2 mL MP108 probiotic (4 × 10^9^ CFU/mL) (Weeks 6–8)	Probiotic group (MP108)

### 2.3. Behavioral Testing

Open‐field test (OFT): The OFT is used to assess mice’s locomotor activity and anxiety levels. The test apparatus consists of an open‐field chamber, a video recording system, and video analysis software (EthoVision XT, Noldus, Netherlands). The open‐field chamber is a white, open‐top square plastic box measuring 50 × 50 × 50 cm. The camera system is positioned directly above the chamber. After adjusting video parameters on the computer, it remains stationary. Experimental recording parameters are configured within the video recording and analysis software. Mice were removed from their housing cages and placed in the center of the open field with their backs toward the experimenter, and the video recording system was immediately activated. Each mouse was allowed to freely explore the open field for 6 min. After the experiment, the mouse was removed and returned to its housing cage. The entire open‐field box was wiped with a 75% ethanol solution and dried with paper towels, and then a new mouse was introduced to repeat the experimental steps. Recording parameters for the OFT include total distance traveled and time spent in the central zone. Motor activity is assessed by total distance traveled, while anxiety levels are evaluated by the ratio of time spent in the central zone to time spent in the peripheral zone. All behavioral data were analyzed by experimenters blinded to the group allocation to minimize observer bias (see Table 2).

**TABLE 2:** Primers for RT‐PCR.

Gene	Primer (5′‐3′)
Gapdh	F‐5′‐ACAACAGCCTCAAGATCGTCAG‐3′
	R‐5′‐ACTGTGGTCATGAGTCCTTCC‐3′

MBP	F‐5′‐ACACACGAGAACTACCCATTATGG‐3′
	R‐5′‐AGAAATGGACTACTGGGTTTTCATC‐3′

Olig2	F‐5′‐GTCTAGTCGCCCATCGTCC‐3′
	R‐5′‐GACACAGTCCCTCCTGTGAAG‐3′

Gfap	F‐5′‐AGCGTGCGGAGATGATG‐3′
	R‐5′‐AGTTTGGTGGGCTCCTTG‐3′

Sox10	F‐5′‐TGGACCGCACACCTTGGGACA‐3′
	R‐5′‐ACGCCCACCTCCTCCGACCT‐3′

Cox2	F‐5′‐CACCCTGACATAGACAGTGAAAG‐3′
	R‐5′‐CTGGGTCACGTTGGATGAGG‐3′

NF‐kB	F‐5′‐GGAGGCATGTTCGGTAGTGG‐3′
	R‐5′‐CCCTGCGTTGGATTTCGTG‐3′

Cxcl1	F‐5′‐CGAGCCACACTCCAACACAGC‐3′
	R‐5′‐AGGGAGCTTCAGGGTCAAGGC‐3′

NOR test: Leveraging mice’s innate tendency to explore novel objects, the NOR test evaluates long‐term memory learning ability in mice under free‐roaming conditions (EthoVision XT, Noldus, Netherlands). Prior to each test, the apparatus is deodorized with 75% alcohol and cleaned. Male and female offspring mice from each group should be tested sequentially. On Test Day 1, mice were acclimated to the apparatus in a 50 × 50 × 50 cm chamber. On Test Day 2, two identical, odorless objects were placed before the mice. Mice freely explored the objects for 10 min, with exploration times recorded. On Test Day 3, an old object (from the second day) and a new object were placed in the same location. Mice freely explored for 5 min. The Supermaze video analysis system recorded and saved the video. Concurrently, researchers who were blinded to the group allocation and experimental conditions manually recorded the offspring mice’s exploration time for the new object, the old object, and the total exploration time for both objects. All behavioral data were analyzed by experimenters blinded to the group allocation to minimize observer bias.

### 2.4. Microbiome Data Analysis

Total DNA was extracted from mouse feces using a fecal extraction kit (MP Biomedicals, Santa Ana, USA). The V3‐V4 region of bacterial 16S rDNA was amplified using primers 341F/806R. PCR conditions include the following: (1) pre‐denaturate at 95°C for 5 min; (2) denature at 95°C for 30 s, anneal at 50°C for 30 s, extend at 72°C for 50 s, and repeat 30 cycles; (3) extend at 72°C for 10 min. PCR products were purified via agarose gel electrophoresis and recovered using the QIAquick Gel Extraction Kit (Qiagen, Dusseldorf, Germany) according to manufacturer instructions. Samples were sequenced on the Illumina MiSeq PE300 platform (Illumina, San Diego, USA). Following initial library sequencing data acquisition, downstream data quality control (QC), assembly, and annotation analyses were performed using QIIME2 software.

### 2.5. Nontargeted Metabolomics Analysis of Feces

Precisely weigh 20 mg of fecal sample into a 2‐mL centrifuge tube. Add 600 μL of methanol and vortex for 30 s. Then, add magnetic beads, place in a tissue grinder, grind at 50 Hz for 120 s, and sonicate at room temperature for 10 min. Centrifuge the sample at 12, 000*g* for 10 min at 4°C. Filter the supernatant using a 0.22 ‐μm membrane filter, transfer the filtrate to a detection vial, and proceed with LC‐MS analysis.

Thermo Vanquish ultra‐high‐performance liquid chromatography system (Thermo Fisher Scientific, Waltham, USA) parameters are as follows: ACQUITY UPLC HSS T3 column (2.1 × 100 mm, 1.8 μm) (Waters Atlantis, Milford, USA); flow rate: 0.3 mL/min; column temperature: 40°C; injection volume: 2 μL; positive ion mode with mobile phases: 0.1% formic acid in water (A1) and 0.1% formic acid in acetonitrile (B1). Gradient elution program: 0–1 min, 8% B1; 1–8 min, 8%–98% B1; 8–10 min, 98% B1; 10–10.1 min, 98%–8% B1; 10.1–12 min, 8% B1. For negative ion mode, the mobile phase consisted of 5 mM ammonium formate in water (A2) and acetonitrile (B2), utilizing the same gradient elution program (replacing B1 with B2). All samples were separated by UHPLC and analyzed by mass spectrometry using a Thermo Orbitrap Exploris 120 mass spectrometer (Thermo Fisher Scientific, Waltham, USA) with electrospray ionization in both positive and negative ion modes for separate scanning. The positive ion spray voltage was 3.50 kV, the negative ion spray voltage was −2.50 kV, the sheath gas was 40 arb, and the assist gas was 10 arb. The capillary temperature was set at 325°C. A full scan at a resolution of 60,000 was performed in the first stage, with an ion scan range of 100–1000 m/z. HCD was employed for secondary fragmentation at a collision energy of 30%, with a secondary resolution of 15,000. The first four ions were collected for fragmentation, while dynamic exclusion was used to eliminate unnecessary MS/MS information.

The raw mass spectrometry data were initially converted into the open mzXML format utilizing the MSConvert module within the ProteoWizard toolkit. Subsequent data processing workflows, which encompassed peak detection, noise filtering, retention time alignment, and peak alignment across all samples, were executed using the RxCMS platform to generate a comprehensive feature table of quantified metabolites. To guarantee data reliability, a stringent quality assessment was conducted based on the reproducibility of the QC samples; specifically, metabolic features were retained only if their relative standard deviation (RSD) values across the QC samples were below 15%. Furthermore, absolute quantification was achieved by constructing calibration curves using a series of standards. For each analyte, the ratio of its peak area to that of the corresponding internal standard was plotted against the concentration, enabling quantification in the samples via linear regression analysis.

### 2.6. Quantitative Real‐Time PCR

Isolate the mouse hippocampus and use trizol to lyse the tissue for RNA extraction. Homogenize with trizol and zirconium beads, incubate for 15 min, then add chilled chloroform, and incubate again. Centrifuge at 12, 000 × *g* for 15 min at 4°C, transfer the aqueous layer, and add isopropanol to precipitate RNA. Centrifuge again, discard the supernatant, and wash the pellet with 75% ethanol. Finally, centrifuge at 7500 × *g* for 5 min at 4°C, retain the pellet, and air‐dry the RNA for 5–10 min. Gently resuspend in DEPC‐treated water and adjust the reverse transcription concentration to 1 μg/μL, ensuring purity (A260/A280) is between 1.8 and 2.0. Use the Vazyme reverse transcription kit (Accurate Biology, Hunan, China) to obtain cDNA and detect it via quantitative PCR, setting up each sample in triplicate. Analyze results with the 2^−ΔΔCt^ method to compare gene expression levels across groups.

### 2.7. Statistical Analysis

All results were expressed as mean ± standard errors, with differences among the groups revealed using one‐way ANOVA. ^∗^ *p* < 0.05, ^∗∗^ *p* < 0.01, and ^∗∗∗^ *p* < 0.001 were considered as significant differences.

## 3. Results

### 3.1. Maternal Obesity Induces Cognitive and Behavioral Abnormalities in Offspring Mice

MP108 intervention and HFD modeling in maternal mice were employed to investigate neurodevelopmental improvements in offspring (Figures 1(a) and 1(b)). By Week 5, significant weight differences emerged between MCD and MHFD maternal groups (*p* < 0.001), confirming successful establishment of the maternal HFD model (Figures 1(c) and 1(d)). Maternal mice were subsequently mated with male mice on a normal diet, maintaining their original dietary regimen throughout gestation and lactation. Findings revealed that offspring from HFD‐fed mothers exhibited significantly increased body weight at 3 weeks of age (*p* < 0.001). However, offspring in the MP108 group reversed this trend, showing no significant weight difference compared to the CD group (Figure 1(e)). The OFT was employed to assess locomotor activity and anxiety levels in offspring. Compared to the CD group, the HFD group showed a significant increase in time spent in the central area of the open field, while the MP108 group reversed this trend (*p* < 0.01, Figure 1(f)). Additionally, compared to the HFD group, MP108 mice exhibited increased time spent exploring a novel object (Figure 1(g)). These results suggest that the probiotic MP108 may contribute to restoring growth and developmental abnormalities in offspring caused by a HFD to some extent.

FIGURE 1Effects of maternal obesity and MP108 probiotic intake on offspring cognitive behavior. Maternal diet and breeding (a), progeny diet (b), maternal prepregnancy weekly body weight (c), maternal body weight at mating (Week 5) (d), progeny body weight at Week 3 (e), progeny time spent in the center zone of the open field (f), and progeny time spent exploring a novel object (g). Data are presented as mean ± standard error of the mean (SEM). ^∗^ *p* < 0.05; ^∗∗^ *p* < 0.01; ^∗∗∗^ *p* < 0.001.

**Figure:**
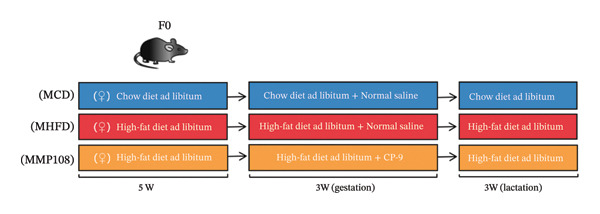
(a)

**Figure:**
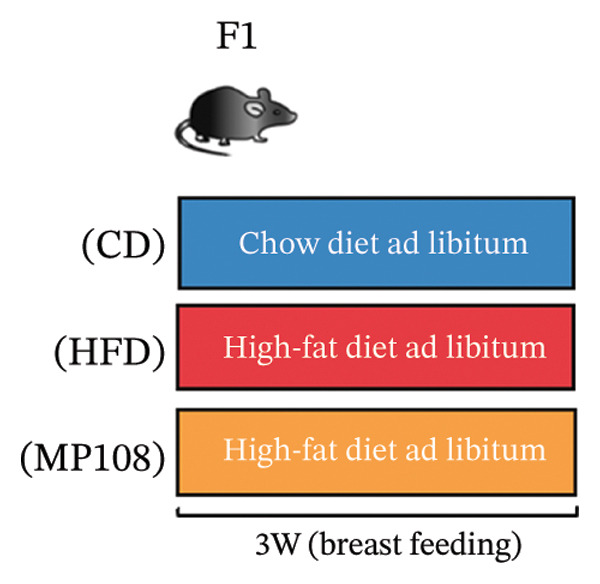
(b)

**Figure:**
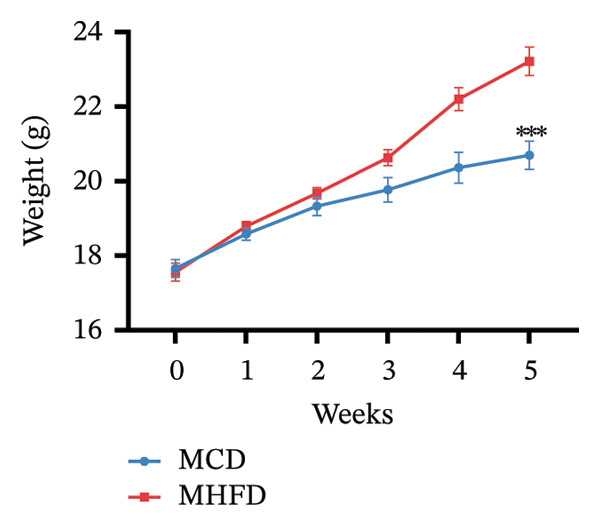
(c)

**Figure:**
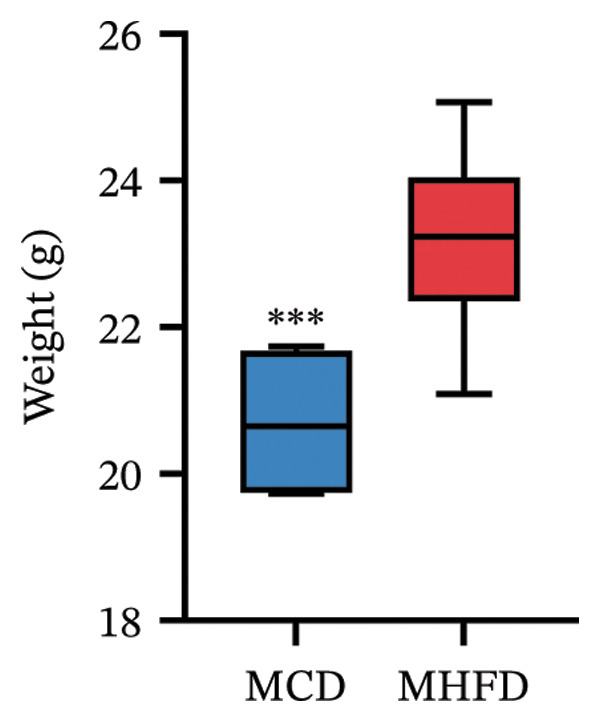
(d)

**Figure:**
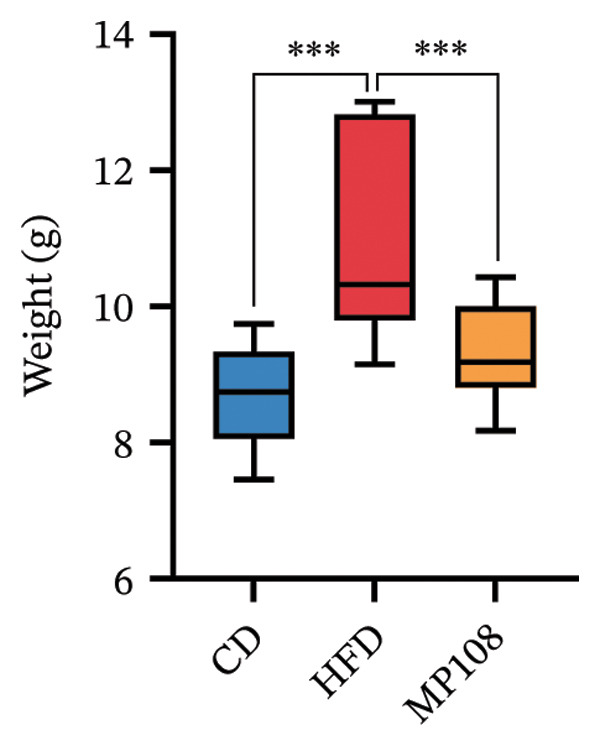
(e)

**Figure:**
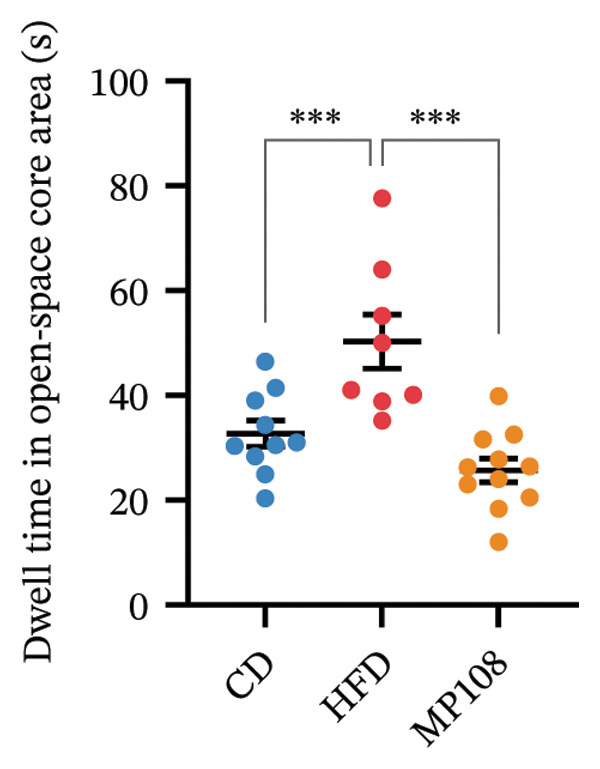
(f)

**Figure:**
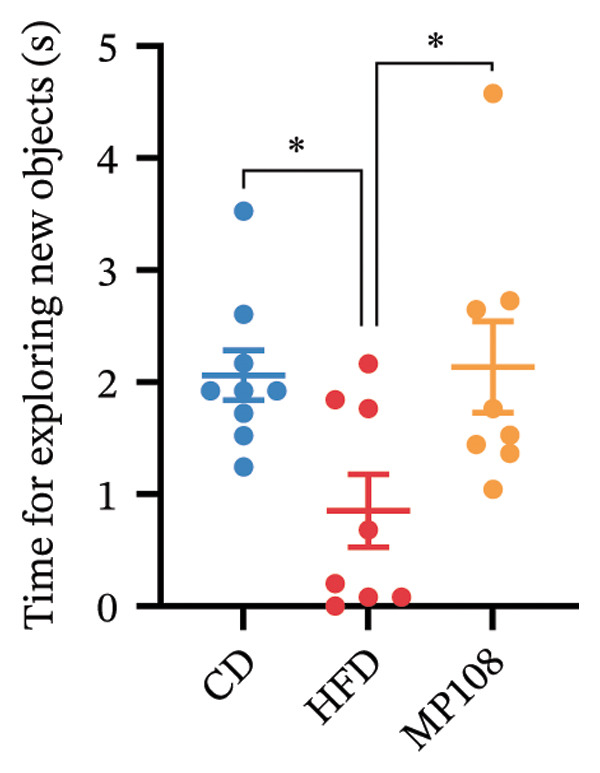
(g)

### 3.2. Maternal Intake of MP108 Reshapes Gut Microbiota

SixteenS rDNA gene sequencing was performed to further analyze the effects of MP108 on maternal gut microbiota. The Chao1 index revealed a significant increase in the MMP108 group, indicating that MP108 intake enhanced species richness in the maternal gut microbiota (Figure 2(a)). Principal component analysis (PCoA) at the species level revealed that the MMP108 group exhibited similar patterns to the MHFD group, while both differed significantly from the MCD group, indicating that the HFD altered the community structure of the gut microbiota (Figure 2(b)). At the phylum level, compared with the MHFD group, the MMP108 group showed a relative decrease in Firmicutes and a relative increase in Bacteroidetes (Figure 2(c)). Among the top 20 most abundant genera, the MMP108 group showed positive correlations with Lactobacillus, Faecalibaculum, Romboutsia, Blautia, and Coriobacteriaceae UCG‐002 and a negative correlation with Enterorhabdus (Figures 2(d), 2(e), 2(f), 2(g), 2(h), 2(i), 2(j), and 2(k)). Further analysis using Lefse intergroup differential species analysis combined with linear discriminant analysis (LDA) revealed differences in genus‐level microbial composition among groups (LDA threshold: 3, *p* < 0.05). Characteristic bacteria for the MMP108 group included Blautia, Romboutsia, and Lactobacillus. Characteristic bacteria for the MHFD group included Ruminiclostridium, Bilophila, and Peptococcus. Characteristic bacteria for the MCD group included Prevotellaceae UCG 001, Adlercreutzia, and Akkermansia.

FIGURE 2MP108 intake in maternal mouse diets improves gut microbiota. α diversity—Chao1 index (a); β diversity—PCoA analysis based on Bray–Curtis (b); gut microbiota composition at phylum level (c); gut microbiota composition at genus level (d); genus‐level correlation heatmap (e); Firmicutes/Bacteroidetes ratio (f); relative abundance of Lactobacillus (g); relative abundance of Blautia (h); relative abundance of Enterorhabdus (i); LEfSe differential species LDA analysis (j); LEfSe differential species cladogram (k). Data are expressed as mean ± standard error of the mean (SEM). ^∗^ *p* < 0.05; ^∗∗^ *p* < 0.01; ^∗∗∗^ *p* < 0.001.

**Figure:**
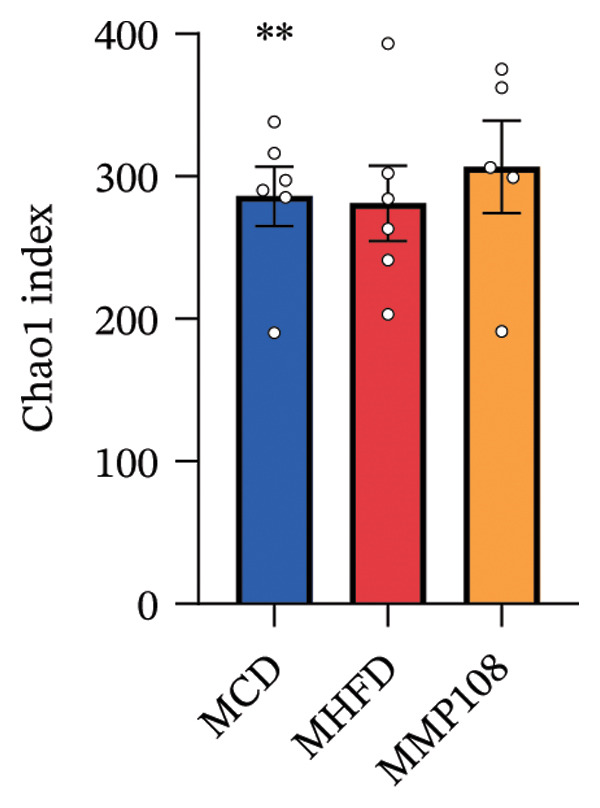
(a)

**Figure:**
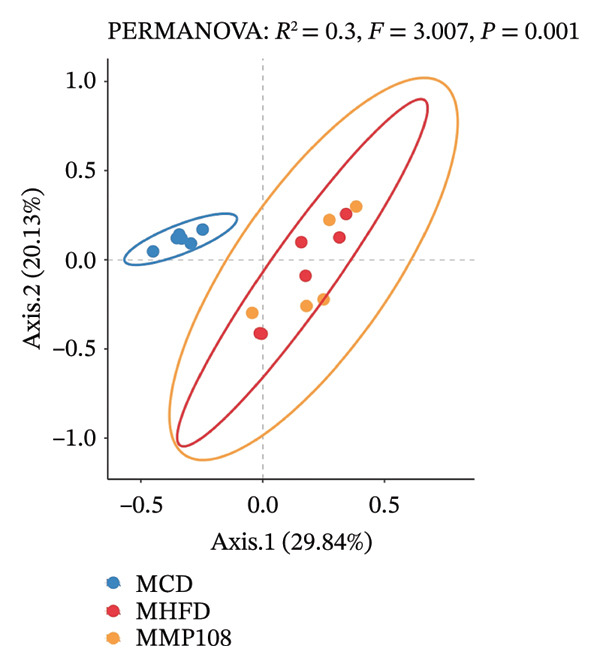
(b)

**Figure:**
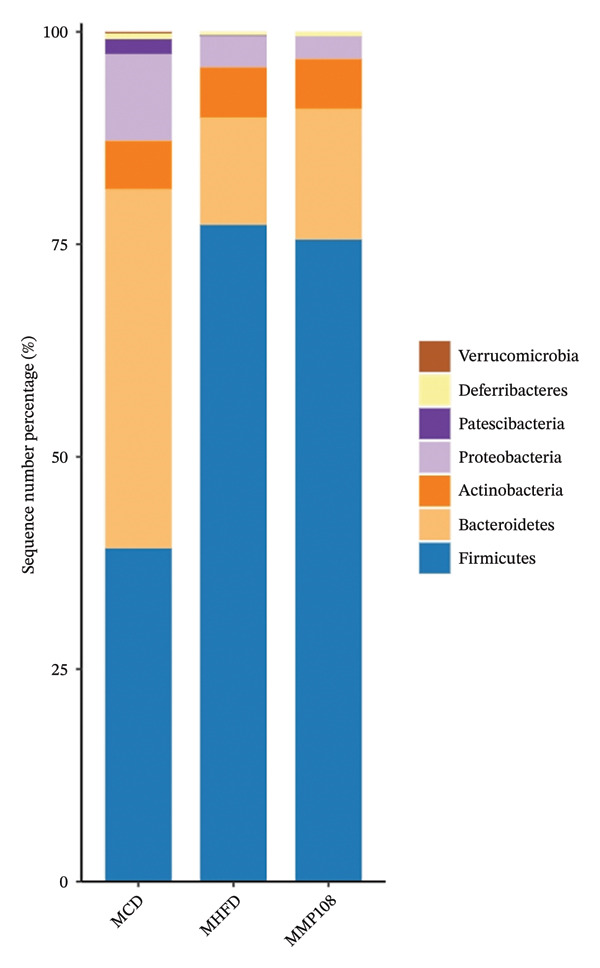
(c)

**Figure:**
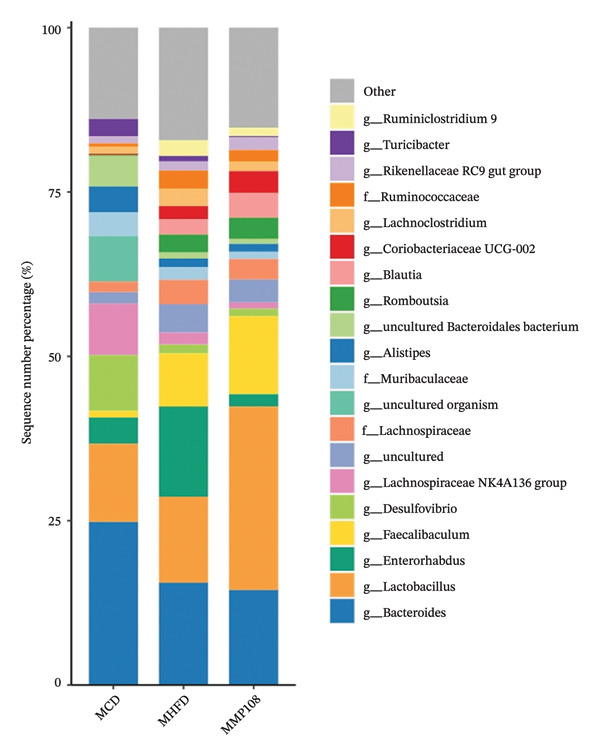
(d)

**Figure:**
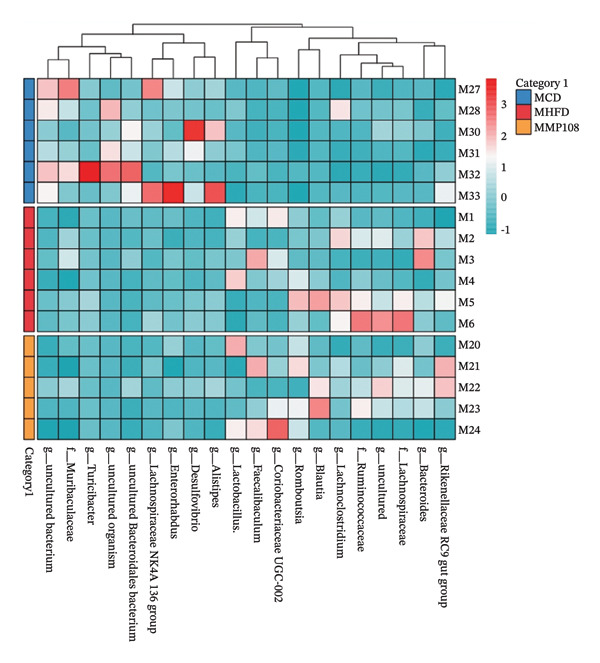
(e)

**Figure:**
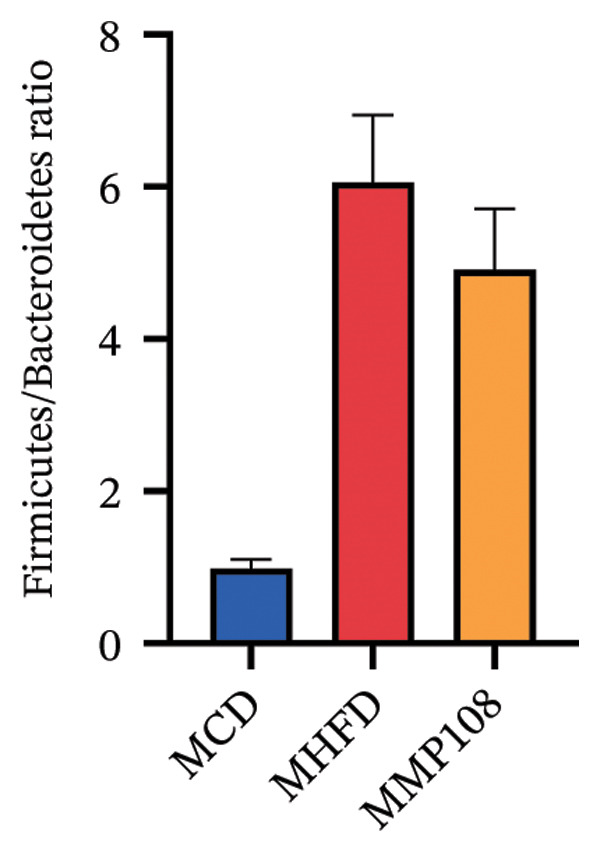
(f)

**Figure:**
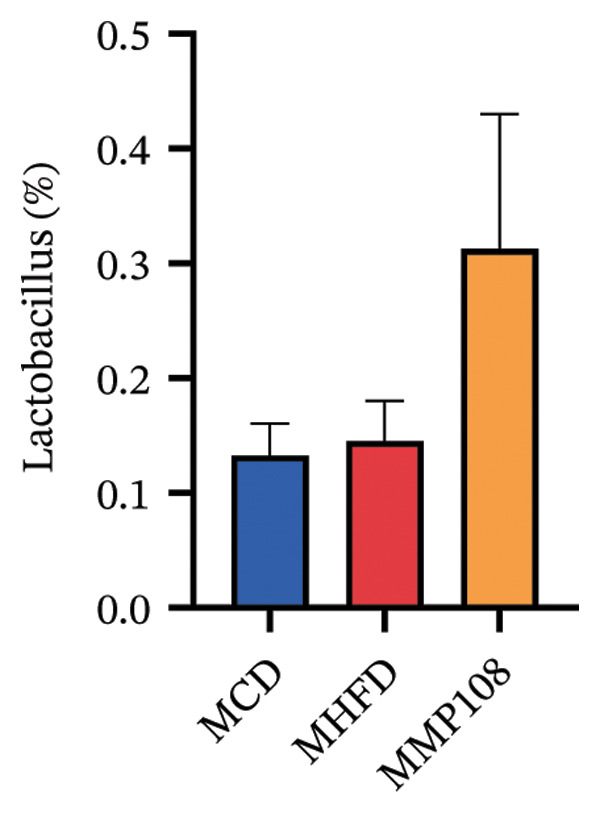
(g)

**Figure:**
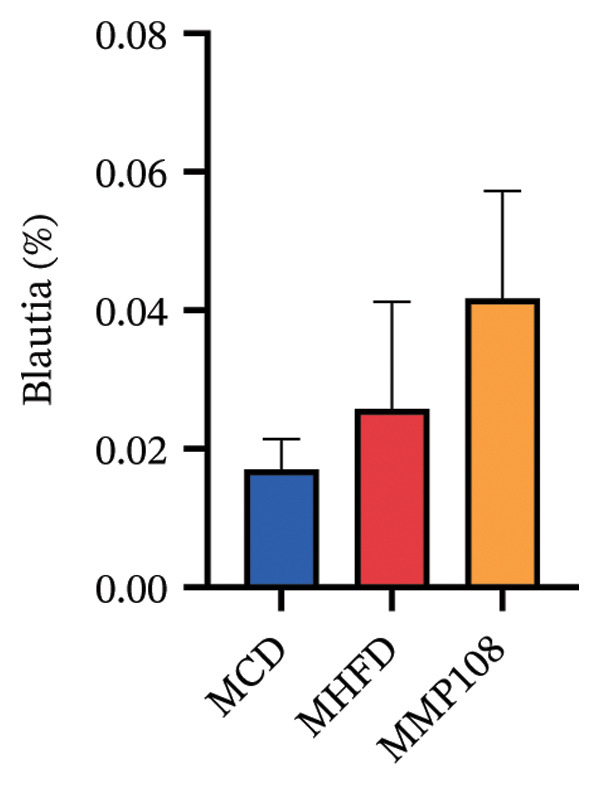
(h)

**Figure:**
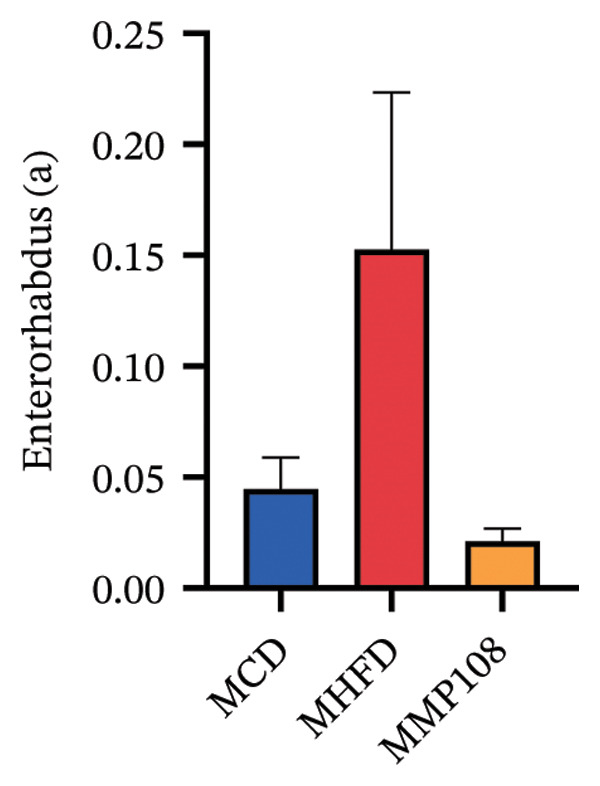
(i)

**Figure:**
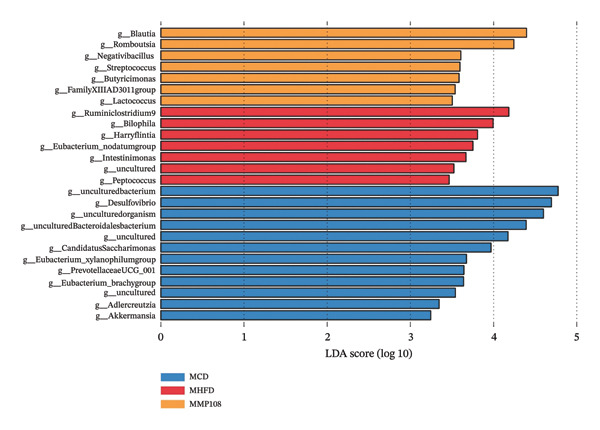
(j)

**Figure:**
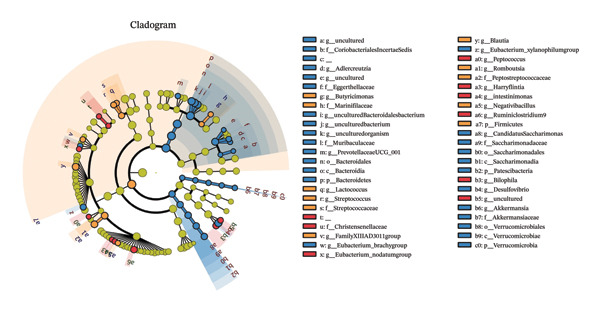
(k)

### 3.3. Breastfeeding Reshapes Offspring Gut Microbiota

Maternal intake of MP108 remodeled gut microbiota abnormalities induced by HFD, thereby further investigating its effects on offspring gut microbiota. Regarding alpha diversity, the Chao1 index and observed index in the CON group were higher than those in the HFD and MP108 groups, indicating that breastfeeding influences offspring gut species richness (Figures 3(a) and 3(b)). Regarding beta diversity, species‐level PCoA and PCA revealed similarities in composition between the HFD and MP108 groups, with distinct differences from the CON group. This indicates that breastfeeding influences offspring gut species composition (Figures 3(c) and 3(d)). Venn diagram analysis of sequence differences revealed 136 shared sequences among the MP108, HFD, and CON groups, with 7, 84, and 3 differentially abundant sequences, respectively (Figure 3(e)). At the phylum level, maternal HFD feeding increased Firmicutes and decreased Bacteroidetes in the HFD offspring compared to the CON group. The MP108 group reversed this trend and showed significant differences compared to the HFD group (*p* < 0.05) (Figure 3(f)). At the genus level, the MP108 group enriched Bacteroidetes, Blautia, Alloprevotella, and Muribaculum compared to both the CON and HFD groups (Figure 3(g)). Correlation heatmaps further confirmed strong associations between these four genera and the MP108 group (Figure 3(h)), with Blautia and Alloprevotella showing significant differences in the MP108 group (Figures 3(j), 3(k), 3(l)). Further analysis using Lefse intergroup differential species analysis combined with LDA (Figures 3(m), 3(n)) revealed differences in genus‐level microbial composition among groups (LDA threshold: 3, *p* < 0.05). Characteristic bacteria of the MP108 group included Bacteroides, Blautia, Romboutsia, Muribaculum, g_RikenellaceaeRc9gutgroup, and PrevotellaceaeUCG 001. Characteristic bacteria of the HFD group included Bilophila, Enterococcus, RuminococcaceaeNK4A214group, Lachnoclostridium, Roseburia, and Ruminococcaceae UCG 010.

FIGURE 3Breastfeeding reshapes the gut microbiota of offspring mice. α‐diversity analysis: Chao1 index (a) and observed index (b); β‐diversity analysis: Bray–Curtis PCoA plot (c); PCA: principal component analysis (d); feature sequence Venn diagram (e); genus‐level gut bacterial composition (f); genus‐level correlation heatmap (g); Firmicutes/Bacteroidetes ratio (h); Lactobacillus relative abundance (i); Lactobacillus relative abundance (j); Blautia relative abundance (k); Enterorhabdus relative abundance (l); LEfSe differential species LDA analysis (m); and LEfSe differential species cladogram (n). Data are expressed as mean ± standard error of the mean (SEM). ^∗^ *p* < 0.05; ^∗∗^ *p* < 0.01; ^∗∗∗^ *p* < 0.001.

**Figure:**
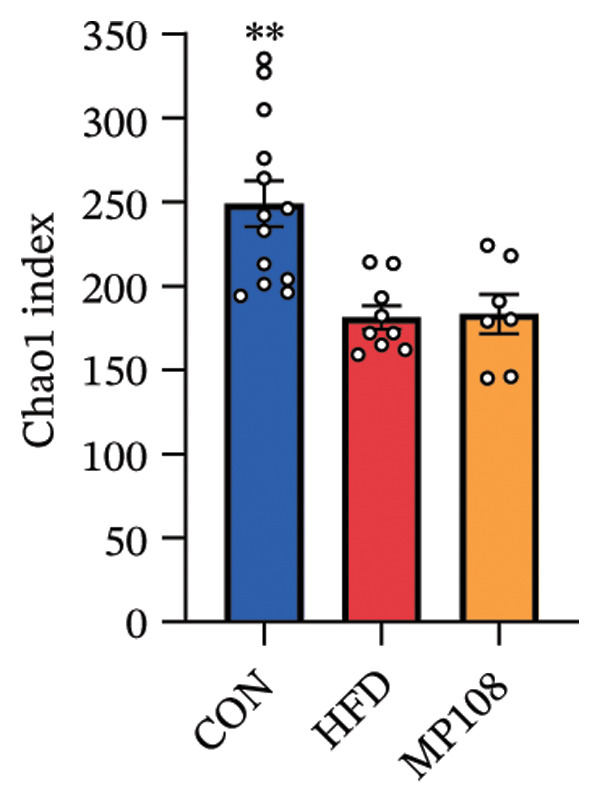
(a)

**Figure:**
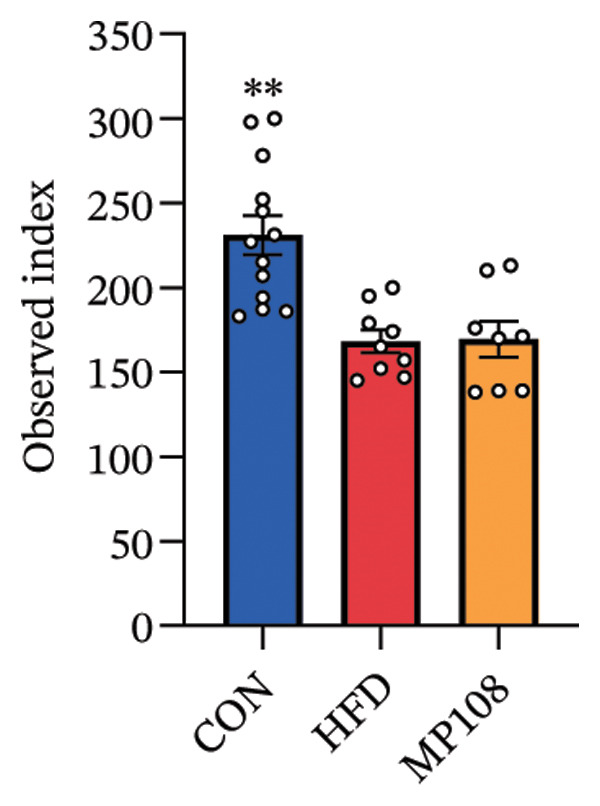
(b)

**Figure:**
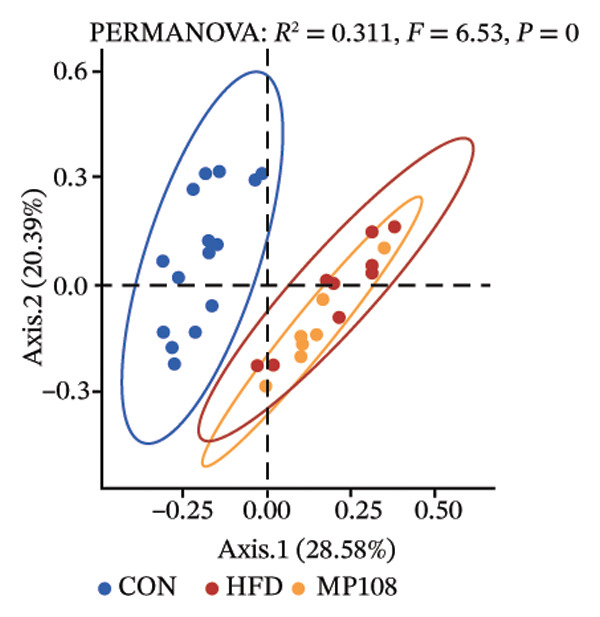
(c)

**Figure:**
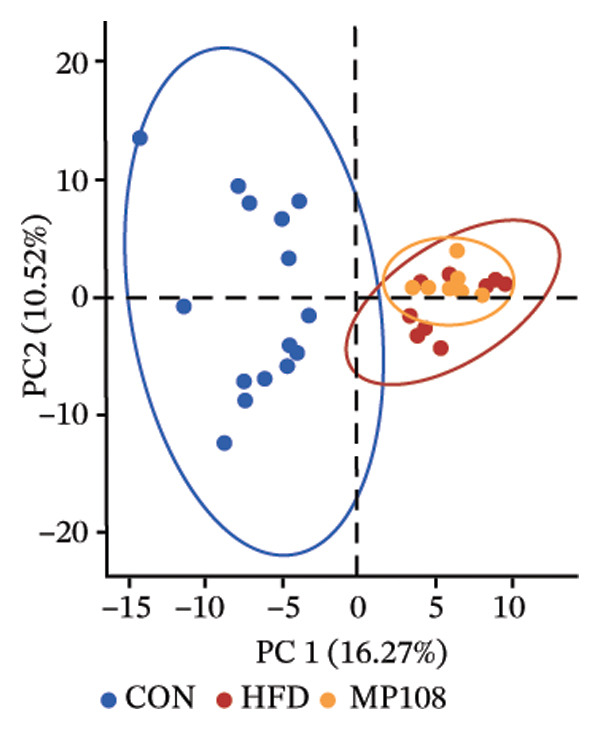
(d)

**Figure:**
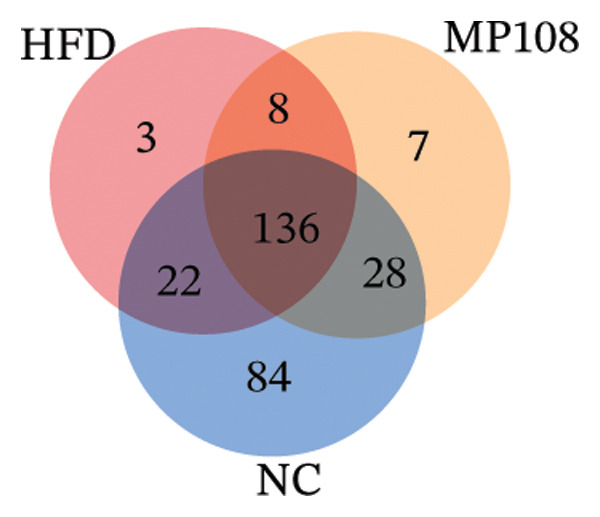
(e)

**Figure:**
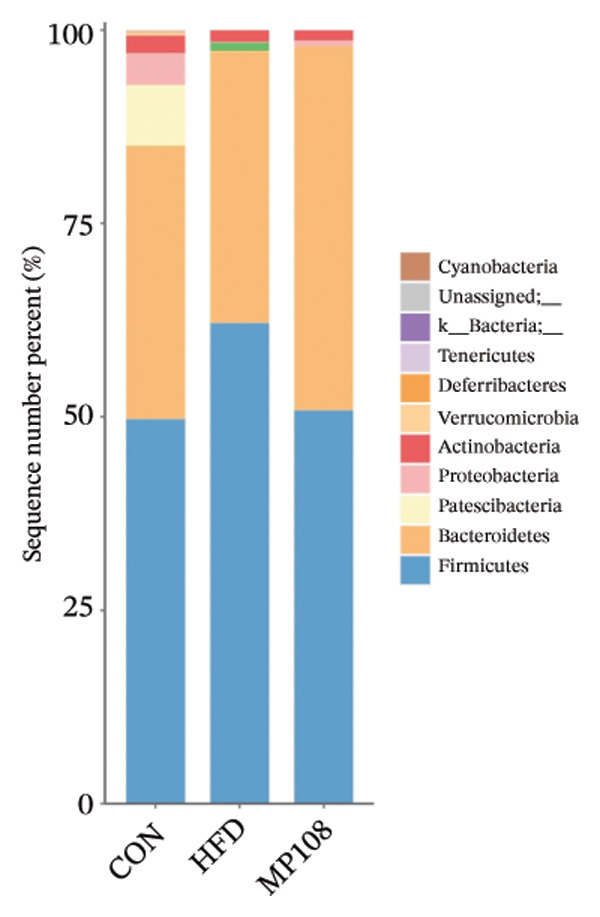
(f)

**Figure:**
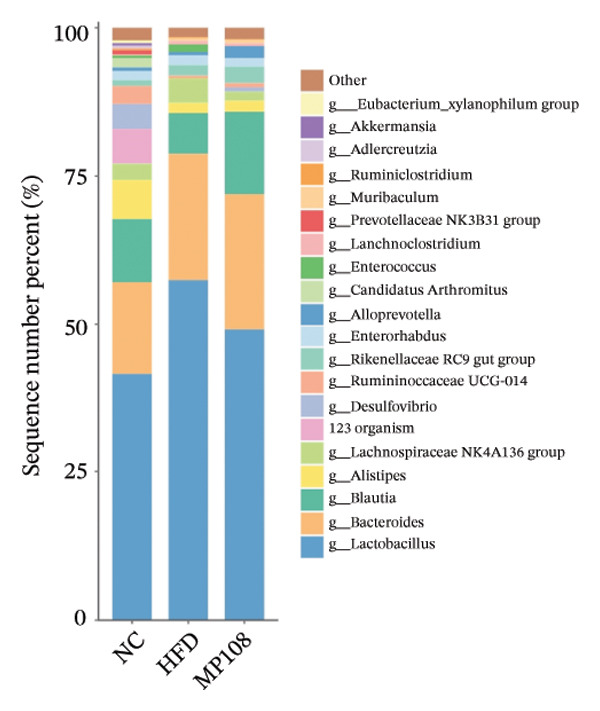
(g)

**Figure:**
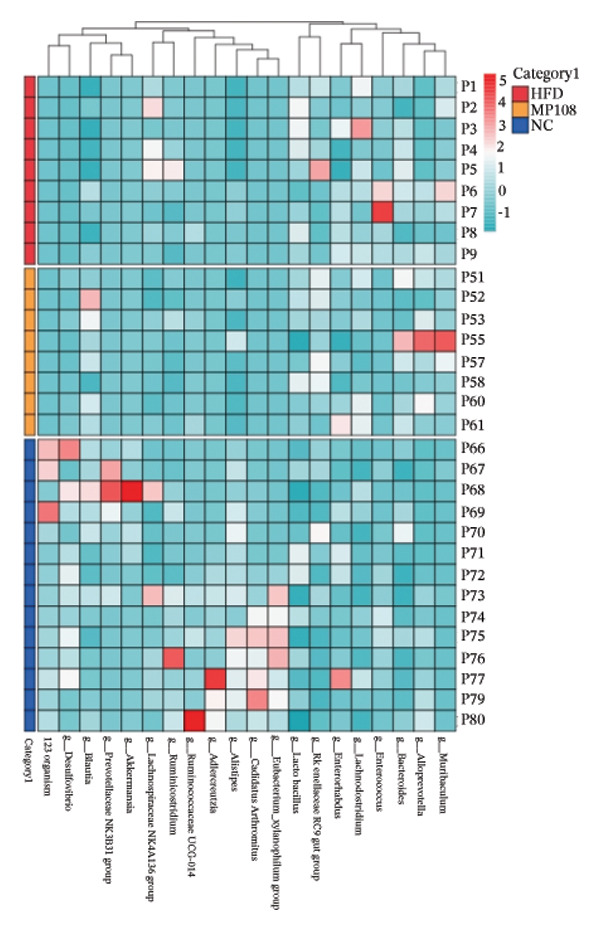
(h)

**Figure:**
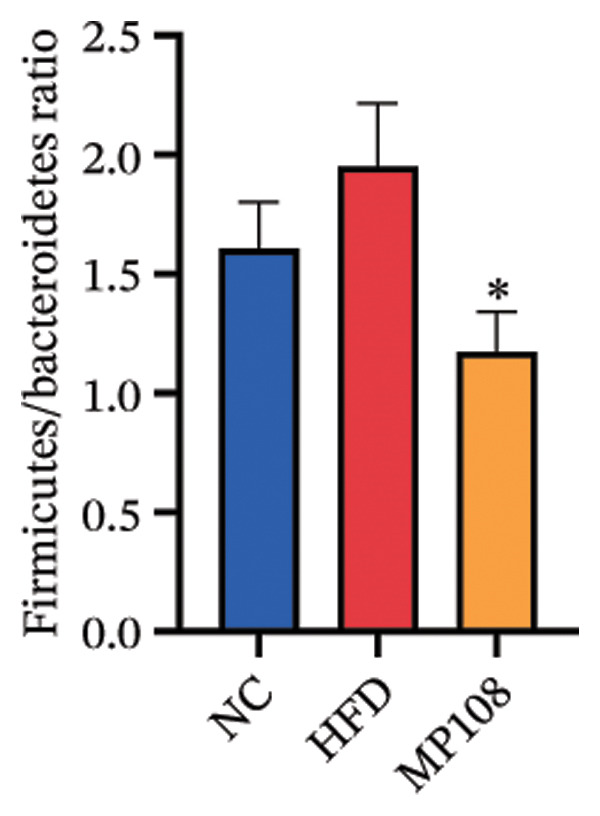
(i)

**Figure:**
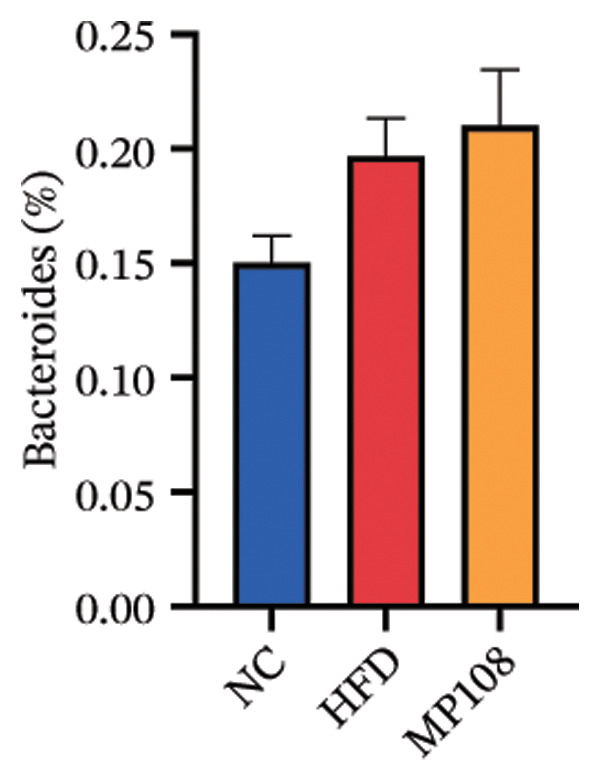
(j)

**Figure:**
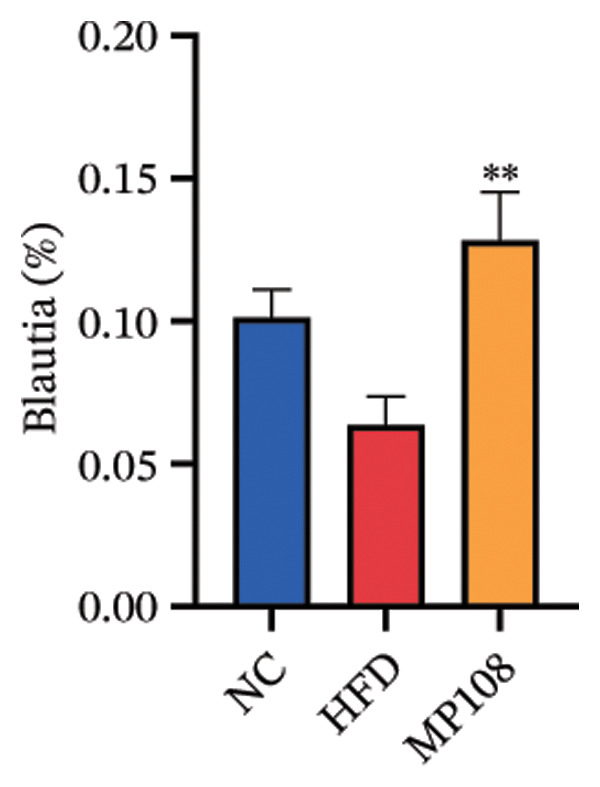
(k)

**Figure:**
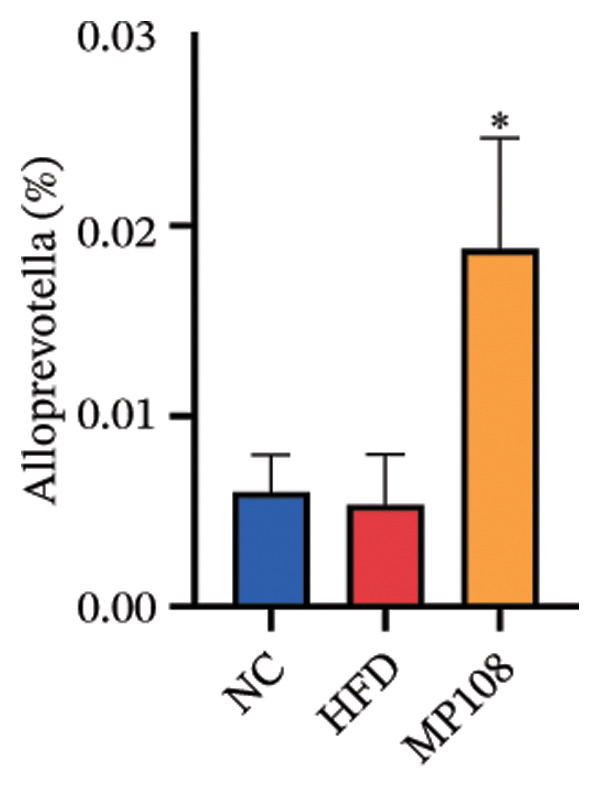
(l)

**Figure:**
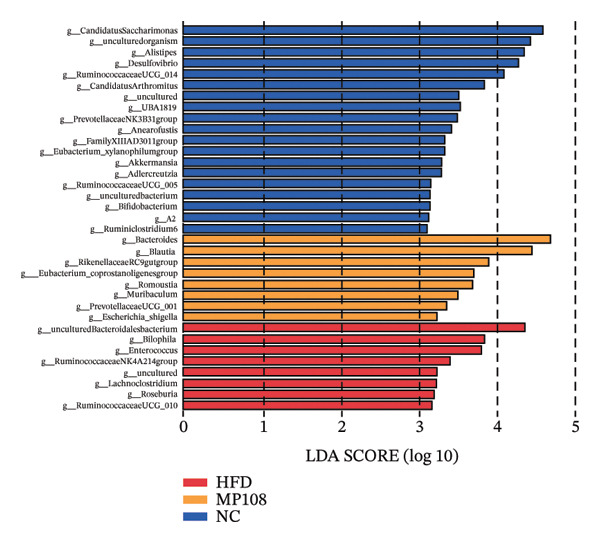
(m)

**Figure:**
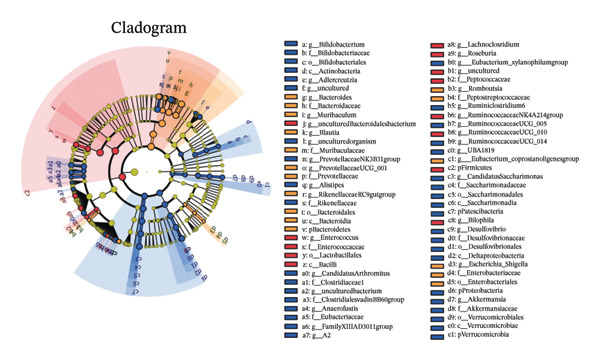
(n)

### 3.4. Metabolome Modulation Improves Offspring Neurodevelopmental Abnormalities Induced by Maternal HFD

To further investigate the effects of probiotic MP108 intervention on maternal and offspring neurodevelopment, nontargeted metabolomics visualized metabolite differences between maternal and offspring groups. Differentially expressed metabolites were identified using FC > 2 and *p* value threshold: 0.1 (raw). Results revealed that the Axis 1 profiles of maternal MHFD and MMP108 groups exhibited similar levels compared to the MCD group, indicating comparable gut metabolite profiles between these two groups (Figure 4(a)). A total of 106 differentially expressed metabolites (47 upregulated and 59 downregulated) were identified between the MCD and MHFD groups (Figure 4(b)), while 6 metabolites (5 upregulated and 1 downregulated) were identified between the MMP108 and MHFD groups (Figure 4(c)). In offspring, the metabolic similarity of the MP108 group approached that of the control group, indicating partial recovery of metabolic status (Figure 4(d)). A total of 28 differentially expressed metabolites (20 upregulated and 8 downregulated) were identified between the CD and HFD groups (Figure 4(e)), while 17 differentially expressed metabolites (12 upregulated and 5 downregulated) were identified between the MP108 and HFD groups (Figure 4(f)). Among maternal differential metabolites, MMP108 significantly increased the abundance of 17‐hydroxykauran‐19‐oic acid, L‐(+)‐ccitrulline, and trans‐10‐heptadecenoic acid compared to the MHFD group (Figure 4(g)). In the offspring differential metabolites, compared with the MHFD group, the MP108 group showed significantly increased abundance of L‐tyrosine, L‐(+)‐citrulline, L‐(+)‐arginine, isoleucine, 2‐hydroxycinnamic acid, 2′‐deoxyguanosine, and 2′‐deoxyadenosine (Figure 4(h)) and significantly decreased abundance of L‐homocystine and methionine sulfoxide. To further investigate the relationship between gut microbiota and metabolites, a bivariate correlation analysis was performed (Figure 4(i)). Blautia showed significant positive correlations with L‐(+)‐arginine and 2′‐deoxyguanosine. Romboutsia exhibited significant positive correlations with L‐(+)‐arginine, isoleucine, L‐tyrosine, 2‐hydroxycinnamic acid, and 2′‐deoxyadenosine. PrevotellaceaeUCG 001 showed significant positive correlations with L‐(+)‐arginine, L‐(+)‐citrulline, L‐tyrosine, 2‐hydroxycinnamic acid, and 2′‐deoxyadenosine, while Bacteroides correlated significantly with L‐(+)‐arginine. Finally, we examined the expression of neurodevelopmental genes in the hippocampal region of offspring. In the HFD group, mRNA levels of MBP, Olig2, and NF‐κB were overexpressed. MP108 intervention significantly reduced the gene expression levels of MBP, Olig2, Sox10, Cox2, and NF‐κB, restoring them to normal levels. No significant differences in Gfap and Cxcl1 expression were observed across all groups.

FIGURE 4MP108 intervention in the maternal generation improves metabolites in the gut of offspring mice. PLS‐DA analysis of differential metabolites in maternal feces (a); T‐test volcano plot analysis of differential metabolites in maternal feces (b); T‐test volcano plot analysis of differential metabolites in maternal feces (c); PLS‐DA analysis of differential metabolites in offspring feces (d); *T*‐test volcano plot analysis of differential metabolites in offspring feces (NC vs. HFD) (e); *T*‐test volcano plot analysis of differential metabolites in offspring feces (MP108 vs. HFD) (f); analysis of differential metabolites in feces between maternal MMP108 and MHFD groups (g); analysis of differential metabolites in MP108 and HFD offspring fecal samples (h); correlation analysis between offspring fecal metabolites and gut microbiota (i); expression of neurodevelopmental genes in the hippocampal region of offspring (j); correlation network diagram of neurodevelopmental genes, metabolites, and gut microbiota in offspring (k). Data are expressed as mean ± standard error of the mean (SEM). ^∗^ *p* < 0.05; ^∗∗^ *p* < 0.01; ^∗∗∗^ *p* < 0.001.

**Figure:**
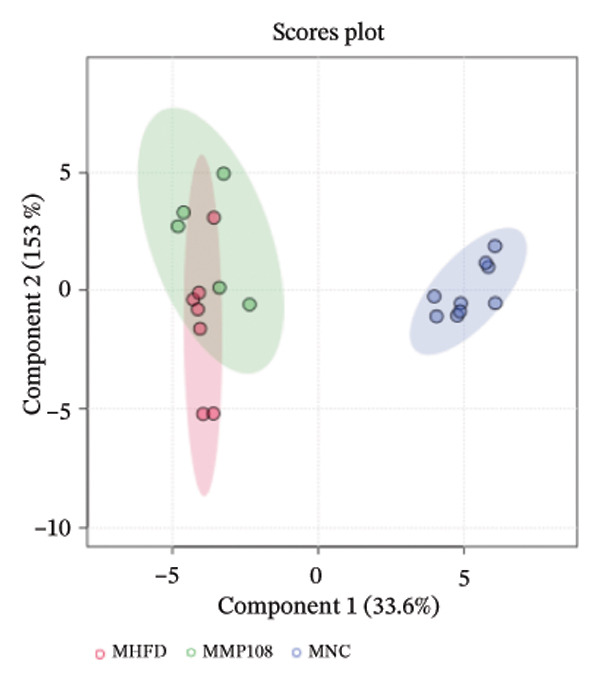
(a)

**Figure:**
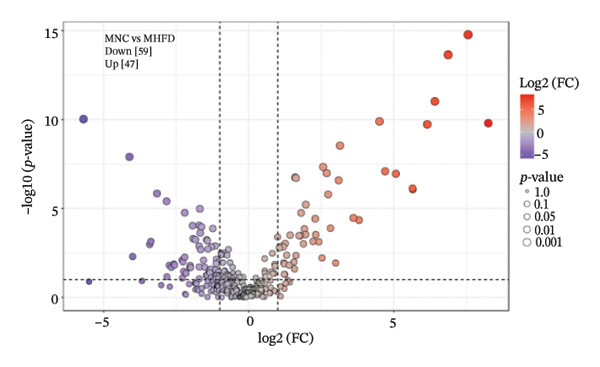
(b)

**Figure:**
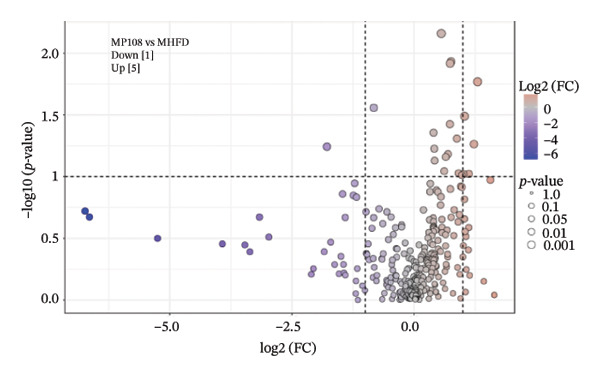
(c)

**Figure:**
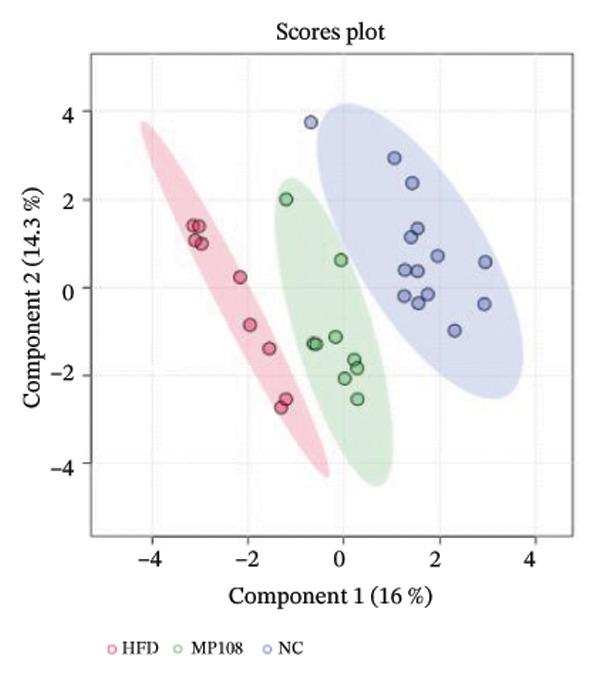
(d)

**Figure:**
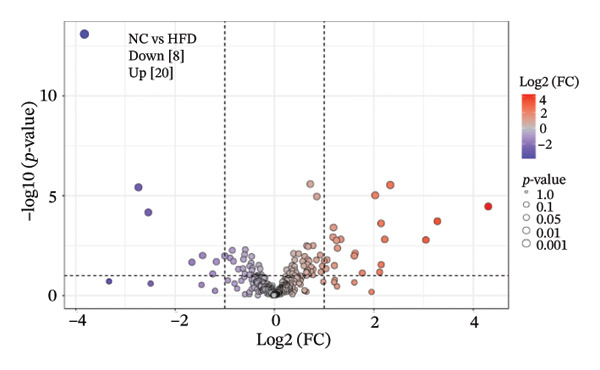
(e)

**Figure:**
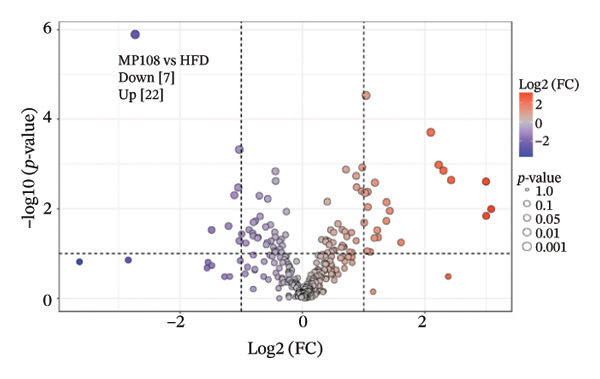
(f)

**Figure:**
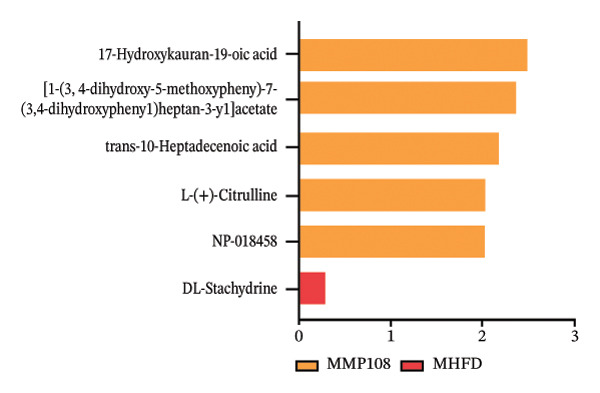
(g)

**Figure:**
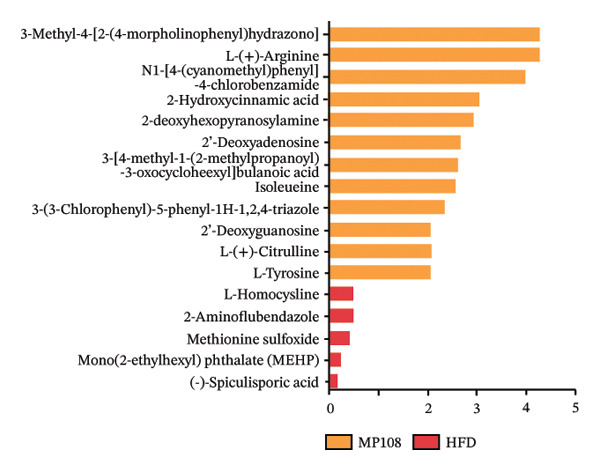
(h)

**Figure:**
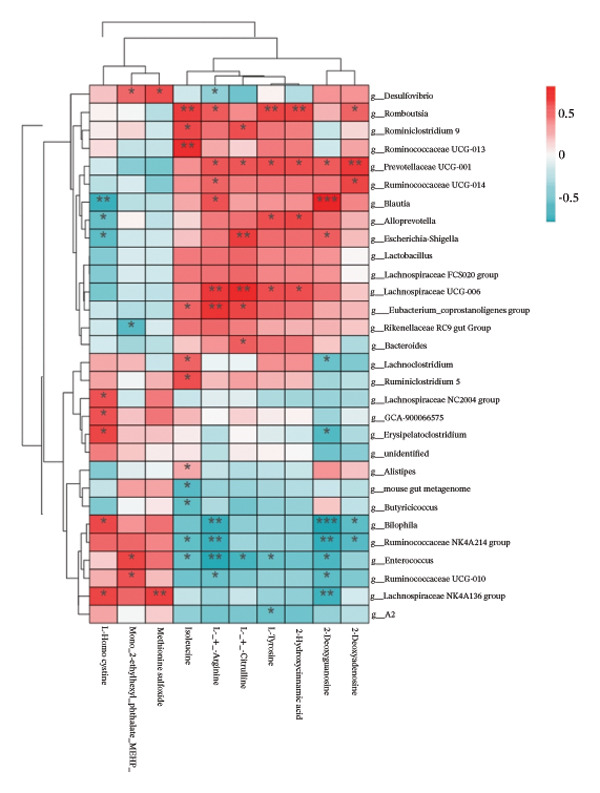
(i)

**Figure:**
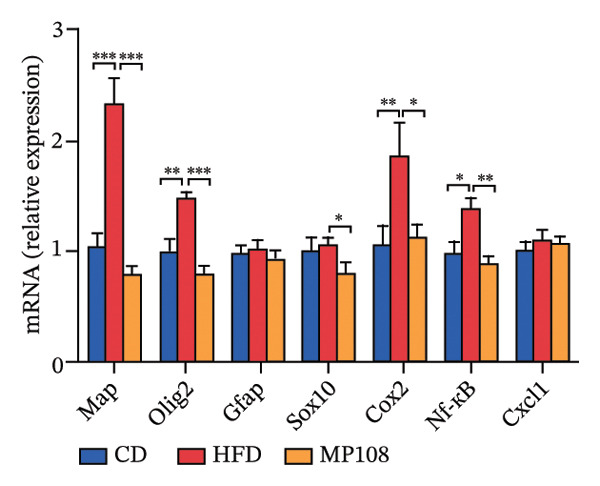
(j)

**Figure:**
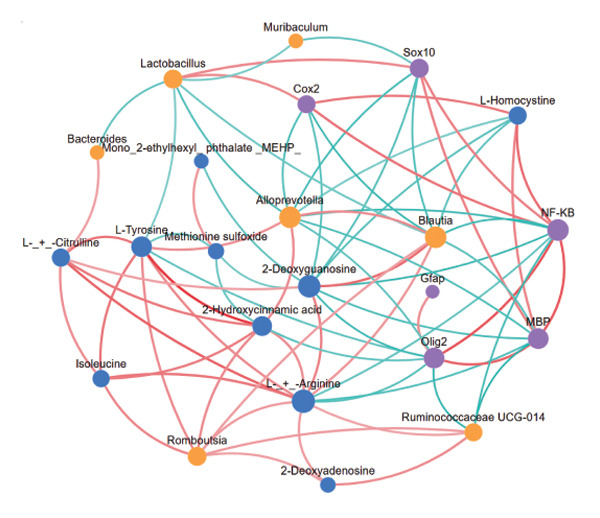
(k)

## 4. Discussion

Obesity, as an increasingly serious public health issue, has drawn significant attention for its impact on offspring. This study systematically investigated the effects of maternal gut microbiota on offspring neurodevelopment by establishing a maternal HFD‐induced obesity model and implementing prenatal intervention with MP108. Results indicate that maternal HFD not only significantly alters its own gut microbiota structure but also influences offspring gut microbial composition through vertical transmission, thereby modifying offspring cognitive behavioral abnormalities and gene expression related to neurodevelopment. Interestingly, while MP108 did not significantly restore the Firmicutes/Bacteroidetes ratio in mothers, it significantly restored this ratio in offspring mice. At the genus level, offspring mice exhibited enrichment of beneficial microbes, including Bacteroidetes, Blautia, Alloprevotella, and Muribaculum. This reduced levels of the harmful metabolite L‐homocysteine and suppressed abnormal expression of genes such as Cox2, NF‐κB, and MBP in the hippocampus, ultimately mitigating the adverse effects of maternal obesity on offspring neurodevelopment.

Previous studies indicate that maternal gut microbiota can influence offspring neurodevelopment through two pathways: lactation and vertical transmission [6]. In this study, the MP108 intervention significantly increased the abundance of Blautia in both maternal and offspring intestines. This bacterium is a key producer of SCFAs, exhibiting anti‐inflammatory and metabolic regulatory effects, and showing a significant negative correlation with L‐homocystine levels (Figure 4(i)) [7]. Research indicates that oral administration of Blautia improves obesity and Type 2 diabetes through gut microbiota metabolism and exhibits anti‐inflammatory effects [8]. Another study observed increased Blautia abundance in the gut microbiota of overweight/obese nonalcoholic fatty liver disease patients following a 3‐week low‐calorie, high‐protein diet [9]. Concurrently, Blautia is a common acetate producer in the gut. It inhibits insulin signaling and fat accumulation in adipocytes by activating G protein‐coupled receptors GPR41 and GPR43, thereby promoting the metabolism of unbound lipids and glucose in other tissues and alleviating obesity‐related diseases [10, 11]. L‐homocystine, an oxidized form of L‐homocysteine, is closely associated with multiple diseases [12]. It disrupts neuronal function through oxidative stress and DNA damage mechanisms, promoting neuroinflammation and neurodegenerative diseases. Its metabolic abnormalities are also linked to neurodegenerative disorders such as Alzheimer’s disease [13]. This study found that the MP108 intervention promoted the proliferation of beneficial bacteria such as Blautia and reduced L‐homocystine production.

By constructing a network diagram linking gut microbiota, metabolites, and genes related to neurodevelopment, significant positive correlations were identified between L‐homocystine and cyclooxygenase‐2 (Cox2), nuclear factor kappa‐B (NF‐κB), and myelin basic protein (MBP) (Figure 4(k)). Cox2, NF‐κB, and MBP are molecules closely associated with neurodevelopment and metabolic regulation, playing crucial roles in normal nervous system function and disease mechanisms [14, 15]. Cox2 activity is closely linked to synaptic signaling, and its overexpression may induce neuroinflammation, thereby affecting neurodevelopment and function [16]. Excessive NF‐κB activation may trigger inflammatory responses and oxidative stress and can also lead to metabolic disorders, neurotoxicity, and neurodegenerative diseases by regulating the expression of metabolism‐related genes [17, 18]. MBP is a crucial structural component for rapid neural signaling; its dysfunction may cause myelin formation disorders, thereby impairing neurodevelopment and nerve conduction [17]. While this study did not directly assess neuroanatomical changes via histological analysis, the observed restoration of hippocampal mRNA expression levels of Cox2, NF‐κB, and MBP provides strong molecular evidence for the neuroprotective effects of MP108. The normalization of MBP expression, in particular, suggests that MP108 intervention likely supports the restoration of myelin integrity and function, which is often compromised in offspring exposed to maternal HFDs. Furthermore, the suppression of neuroinflammatory markers (Cox2 and NF‐κB) implies a preservation of the neuronal microenvironment conducive to proper synaptic plasticity. Therefore, we propose that MP108 administration restores myelin formation and nerve conduction function at a molecular level, thereby improving offspring neurodevelopment. Future studies are warranted to validate these molecular findings with structural evidence, such as quantifying myelin thickness via electron microscopy, assessing neuronal density via Nissl staining, or evaluating synaptic plasticity markers like PSD95 and synaptophysin.

Beyond L‐homocystine, this study also revealed that MP108 intervention significantly improved levels of multiple other metabolites in the offspring gut, which similarly play crucial roles in neurodevelopment and inflammatory regulation. For instance, post MP108 intervention, offspring feces exhibited markedly elevated amino acid metabolites, including L‐(+)‐arginine, L‐(+)‐citrulline, L‐tyrosine, and isoleucine. These amino acids serve not only as fundamental units for protein synthesis but also participate in neurotransmitter synthesis, antioxidant defense, and immune regulation [19, 20]. Notably, L‐(+)‐arginine, as a precursor of nitric oxide (NO), promotes neurogenesis and synaptic plasticity via the NO‐cGMP signaling pathway [21]. Its elevated levels showed a significant positive correlation with the abundance of probiotics such as Blautia and Romboutsia, suggesting that MP108 may enhance the bioavailability of these beneficial amino acids by promoting the growth of specific probiotics. Furthermore, MP108 intervention significantly increased levels of nucleotide metabolites such as 2′‐deoxyguanosine and 2′‐deoxyadenosine in the offspring’s gut. These nucleotides serve as crucial precursors for DNA synthesis and repair; their elevated levels may help mitigate HFD‐induced DNA damage and promote neural cell proliferation and differentiation [22]. Notably, 2′‐deoxyguanosine is also closely associated with antioxidant functions, mitigating oxidative stress damage to the nervous system by scavenging free radicals [23]. This aligns with the observed inhibitory effects of MP108 on oxidative stress‐related genes such as Cox2 and NF‐κB in this study. Another class of metabolites worthy of attention is SCFAs, including acetate, propionate, and butyrate. Although SCFA levels were not directly measured in this study, the MP108 intervention significantly increased the abundance of SCFA‐producing bacteria such as Bacteroidetes, Blautia, Alloprevotella, and Muribaculum in the offspring gut. This suggests SCFAs may participate as key metabolic mediators in regulating neurodevelopment [24, 25]. Future studies will focus on directly quantifying SCFA levels in both maternal and offspring samples to validate this proposed mechanism and further elucidate the functional role of the gut microbiota in mediating the neuroprotective effects of MP108.

MP108 intervention also reduced the levels of methionine sulfoxide in the offspring gut. This compound, a product of methionine oxidation, is closely associated with oxidative stress and neurodegenerative diseases [26]. The reduction in methionine sulfoxide likely reflects MP108’s mitigating effect on oxidative stress, which, together with the decrease in L‐homocysteine levels, constitutes the metabolic regulatory network through which MP108 improves neurodevelopment.

This study also identified enriched genera such as Alloprevotella and Muribaculum in offspring intestines, which are similarly associated with anti‐inflammatory and metabolic regulation. This suggests MP108 may modulate gut microbiota homeostasis through multistrain synergistic effects, thereby influencing offspring neurodevelopment [27]. This finding not only enriches our understanding of the regulatory mechanisms of the microbiota–gut–brain axis in neurodevelopment but also provides new insights for probiotic interventions in maternal and infant health. However, this study has certain limitations. Although correlation analyses revealed associations between bacteria, metabolites, and neurodevelopmental genes, the specific molecular mechanisms require further validation through experiments such as bacterial transplantation and gene knockout. Furthermore, whether MP108 influences neurodevelopment through alternative metabolic pathways (e.g., SCFAs and tryptophan metabolism) warrants further investigation.

In summary, this study confirms that maternal MP108 intervention during pregnancy intergenerationally improves the levels of Blautia and Alloprevotella in offspring, reduces levels of metabolites such as L‐homocystine, methionine, and methionine sulfoxide, increases levels of L‐(+)‐arginine, L‐(+)‐citrulline, L‐tyrosine, and isoleucine, suppresses neuroinflammation, and improves offspring neurodevelopment. Translating our findings from murine models to humans requires careful consideration of interspecies differences in gut microbiota. While the murine dose corresponds to a safe human equivalent dose, optimal dosing for pregnant women needs further validation. Future randomized, double‐blind, placebo‐controlled clinical trials are essential to confirm the safety and efficacy of MP108 for improving maternal and offspring health.

This finding provides experimental evidence for the application of probiotics in preventing and treating offspring neurodevelopmental disorders caused by maternal metabolic abnormalities, laying a theoretical foundation for future functional food development and clinical intervention strategies. Future research may further explore the specific signaling pathways of MP108 and its metabolites in neurodevelopment, offering additional scientific support for precision interventions in maternal and infant health.

## Funding

This study was supported by National Center of Technology Innovation for Dairy (No. 2023‐KFKT‐11).

## Ethics Statement

The study was approved by the Ethics Committee of the Experimental Animal Management Center at Jiangnan University (Approval No.: JN.NO20240315c0450825[083]).

## Conflicts of Interest

The authors declare no conflicts of interest.

## Practical Application

This study found that supplementing with specific probiotics during pregnancy can effectively mitigate the adverse effects of maternal unhealthy diets on offspring brain development. This provides scientific evidence for developing novel probiotic products to support maternal health and promote normal neurodevelopment in infants and young children.

## Data Availability

The data that support the findings of this study are available from the corresponding author upon reasonable request.
